# Primary stability of dental implants placed in low-density polyurethane blocks: Undersized osteotomy versus osseodensification, an in vitro comparative study

**DOI:** 10.4317/medoral.28075

**Published:** 2026-06-28

**Authors:** Michele Agostini, María Dolores Gómez-Adrián, Javier Barberá Millán

**Affiliations:** 1 Universidad Católica de Valencia San Vicente Mártir, Valencia, Spain; 2 Department of Surgery, Universidad Católica de Valencia San Vicente Mártir, Valencia, Spain

**Keywords:** Dental implants, primary stability, low-density bone, undersized osteotomy, osseodensification, resonance frequency analysis, insertion torque.

## Abstract

**Background:**

Primary stability is a determinant of early implant success and becomes critical in low-density bone, where reduced trabecular support may increase micromotion risk. Undersized osteotomy and osseodensification are two commonly discussed site-preparation strategies aimed at improving initial stability through different biomechanical mechanisms.

**Materials and Methods:**

An analytical experimental in vitro study was performed using 76 cylindrical implants (4.0×10mm) (n=38 per group) placed in type IV polyurethane blocks with a dense cortical shell. Group OS underwent an undersized osteotomy drilling sequence, while Group OD underwent osseodensification using Densah® burs operated in counterclockwise rotation. In Group OD, the final osteotomy diameter was intentionally overprepared by 0.3mm (4.3mm) relative to the implant diameter. Maximum insertion torque (Ncm) was recorded during placement. Primary stability was measured as implant stability quotient (ISQ) using the Penguin® resonance frequency analysis device (two perpendicular readings per implant; mean value used). Between-group comparisons were performed using Student’s t-test for independent samples (α=0.05).

**Results:**

Group OS showed higher mean insertion torque than Group OD (30.66±1.71 vs 26.58±2.63 Ncm; p<0.0001). Group OD showed higher mean ISQ than Group OS (56.80±2.90 vs 52.92±3.57; p=0.00000227).

**Conclusions:**

Undersized osteotomy increased insertion torque, consistent with higher rotational resistance during insertion, whereas osseodensification increased ISQ, suggesting higher construct stiffness. Both protocols may be clinically useful in low-density bone depending on the primary objective (torque-driven mechanical engagement vs RFA-derived stiffness).

## Introduction

Dental implant therapy is a predictable treatment option for oral rehabilitation; however, the early mechanical conditions at placement remain essential to achieve stable osseointegration [1,2]. Primary stability is defined as the mechanical engagement between an implant and its surrounding substrate immediately after insertion [3-5] and depends on implant macrogeometry, bone density, surgical technique, and operator-related factors. Inadequate primary stability may lead to increased micromotion at the bone-implant interface, potentially compromising healing, especially in scenarios where early or immediate loading is planned.

Low-density bone is a frequent clinical challenge, particularly in posterior maxillae, where trabecular architecture may provide reduced mechanical support [4,6]. In such situations, clinicians often modify site preparation to enhance initial fixation. Undersized osteotomy is a widely used concept in which the final osteotomy diameter is deliberately prepared smaller than the implant diameter, increasing frictional resistance and lateral compression during insertion [7,8]. This approach may improve insertion torque but can also increase compressive strain if the mismatch is excessive.

Osseodensification is a non-subtractive drilling concept that aims to compact trabecular structure by operating specialized burs in a densifying mode. Rather than removing bone particles, the technique is intended to plastically deform and densify the osteotomy walls and to autograft bone chips into the prepared site. *In vitro* and clinical investigations have suggested that osseodensification may improve resonance frequency-based stability metrics and potentially influence peri-implant bone density [9-12].

Different measurement methods capture distinct aspects of primary stability. Insertion torque reflects rotational resistance during placement and is influenced by osteotomy preparation, implant design, and substrate properties. Resonance frequency analysis (RFA) expresses stability as an implant stability quotient (ISQ) [13,14] and is related to the stiffness of the implant-substrate complex. Because insertion torque and ISQ represent complementary mechanical constructs, two techniques may affect these parameters differently in the same substrate.

The objective of the present study was to compare the primary stability of dental implants placed in a standardized low-density polyurethane model using two different osteotomy preparation protocols: Undersized osteotomy (OS) and osseodensification (OD). Primary stability was assessed by maximum insertion torque and ISQ values measured using the Penguin® RFA system. The null hypothesis was that no significant differences would exist between the two protocols for either outcome.

## Materials and Methods

*Study design and setting*

This study was designed as an analytical experimental *in vitro* investigation to evaluate primary stability of dental implants placed in a standardized synthetic model. The experimental procedures and measurements were performed under controlled conditions.

*Variables*

The independent variable was the osteotomy preparation technique with two levels: Undersized osteotomy (Group OS) and osseodensification (Group OD). The dependent variables were maximum insertion torque (Ncm) and primary stability expressed as ISQ obtained by RFA. To reduce variability, the following factors were kept constant across all samples: Implant design and dimensions, insertion depth (10mm), substrate type, drilling motor settings, implant spacing and ISQ measurement device (Figure 1).

*Synthetic bone model*

Type IV polyurethane blocks (Bone Models® S.L.U., Castellón de la Plana, Spain) were used to simulate low-density cancellous bone, combined with a high-density cortical shell to represent cortical bone. [15,16] Each block measured 22×144×28 mm and allowed multiple implant placements. Osteotomy sites were spaced at least 5 mm apart to minimize mechanical interference between adjacent sites (Figure 2).

*Implants*

Cylindrical implants from the Klockner Vega® system (4.0mm diameter, 10mm length) were used for all samples. The same implant design and dimensions were used in both experimental groups.

*Drilling equipment*

Osteotomies were performed using an NSK Surgic Pro surgical motor equipped with a 20:1 contra-angle. The drilling speed was standardized at 1200 rpm for all preparations. All osteotomies were performed by the same operator following the planned drilling sequences for each group.

*Group allocation*

A total of 76 implants were included and equally allocated to Group OS (n=38) and Group OD (n=38). Osteotomies were prepared sequentially on the blocks, and implants were inserted to the epicrestal level according to the manufacturer’s recommendations.

*Osteotomy preparation protocols*

In Group OS, an undersized osteotomy sequence was performed using the Surgical Box Klockner Vega kit as follows: 2.35mm lance-shaped drill, 2.35mm pilot drill, 2.8mm twist drill, 4.0mm countersink drill, 3.3mm twist drill and 3.6mm twist drill (Figure 3).

In Group OD, the initial drilling was performed with a 2.35mm lance-shaped drill and a 2.35mm pilot drill up to 10 mm depth. Subsequently, osseodensification burs (Densah® burs, Versah®, Jackson, MI, USA) were used in counterclockwise rotation in the following sequence: 2.0mm, 2.3mm, 3.0mm, 3.3mm, 4.0mm and 4.3mm. The final osteotomy diameter in Group OD (4.3mm) represented an intentional overpreparation of 0.3mm relative to the implant diameter (4.0mm). This small overpreparation was chosen to moderate peak compressive strain while still allowing lateral compaction of trabecular elements at the osteotomy margins.

*Implant placement and insertion torque measurement*

Implants were inserted using the calibrated surgical motor. The insertion torque was initiated at 15 Ncm and increased gradually in 5 Ncm increments as required until complete insertion was achieved. The maximum torque value reached during placement was recorded as the insertion torque for the sample.

*Resonance frequency analysis (ISQ)*

Primary stability was further assessed using the Penguin® RFA device (Integration Diagnostics, Sweden). A MultiPeg™ type 26 was attached to each implant, and the probe was positioned close to the peg without contact. Two ISQ measurements were obtained for each implant in perpendicular directions (buccolingual and mesiodistal), and the final ISQ value was calculated as the arithmetic mean of both measurements [13,14].

*Sample size calculation*

Sample size was estimated using power analysis for two independent means based on prior *in vitro* investigations evaluating differences in insertion torque and ISQ under low-density conditions. A minimum clinically relevant difference of 10 Ncm for insertion torque and 6 ISQ points was used, with α=0.05 and power of 80%. The calculation yielded 36 implants per group for insertion torque and 28 implants per group for ISQ. To ensure robustness and account for potential exclusions, the final sample consisted of 38 implants per group (76 total), and all samples were analyzed [12,15,16].

*Statistical analysis*

Data were recorded in a spreadsheet and analyzed using descriptive statistics (mean±standard deviation). Between-group comparisons were performed using Student’s t-test for independent samples. Within-group correlations between insertion torque and ISQ were assessed using the Pearson correlation coefficient. Statistical significance was set at *p<*0.05.

Ethical considerations. The study was conducted exclusively *in vitro* using commercially available synthetic materials; therefore, no human participants or animals were involved and ethics committee approval was not required.

## Results

A total of 76 implants were evaluated, with 38 implants per experimental group. Descriptive statistics for insertion torque and ISQ are summarized in Table 1 and graphically presented in Figure 4.

*Insertion torque*

Group OS exhibited a higher mean insertion torque compared with Group OD (30.66±1.71 Ncm vs 26.58±2.63 Ncm). The difference between groups was statistically significant (*p<*0.0001). The mean difference (OS-OD) was 4.08 Ncm (95% CI:3.05 to 5.11), representing a large effect size (Hedges’ g=1.84). Group OS showed lower standard deviation values, indicating less variability in rotational resistance.

*Resonance frequency analysis (ISQ)*

Group OD showed higher ISQ values compared with Group OS (56.80±2.90 vs 52.92±3.57). This difference was statistically significant (*p=*0.00000227). The mean difference (OD-OS) was 3.88 ISQ units (95% CI: 2.46 to 5.30), corresponding to a large effect size (Hedges’ g=1.21). ISQ variability was comparable between groups, with slightly narrower dispersion in Group OD.

*Correlation between insertion torque and ISQ*

Within-group Pearson correlation analysis did not reveal a relevant linear association between insertion torque and ISQ in either the OS group (n=38; r=-0.10; *p=*0.54) or the OD group (n=38; r=-0.23; *p=*0.16).

## Discussion

The present *in vitro* study compared two implant site preparation protocols-undersized osteotomy and osseodensification-in a standardized low-density polyurethane model. The main finding was that the techniques influenced primary stability outcomes differently depending on the metric considered: Undersized osteotomy increased insertion torque, whereas osseodensification increased ISQ values [12,15,16].

Higher insertion torque in Group OS can be explained by the reduced osteotomy diameter, which increases frictional resistance and lateral compression during implant insertion. This mechanical engagement may be advantageous when the clinical goal is to maximize rotational stability at placement. However, torque values should be interpreted cautiously, as excessive compressive strain could be detrimental in living bone due to microdamage and impaired vascularization [7,8].

Osseodensification yielded higher ISQ values in Group OD, suggesting a stiffer implant-substrate complex under RFA assessment. This may reflect improved contact conditions and compaction of the surrounding trabecular structure produced by densifying burs. Importantly, in Group OD the final osteotomy was intentionally overprepared by 0.3mm (4.3mm for a 4.0mm implant). Osseodensification is designed to compact and laterally displace trabecular bone, increasing peri-implant apparent density and stiffness; this mechanism presupposes the presence of deformable cancellous structures that can be plastically displaced. In contrast, cortical bone is dense and less amenable to plastic compaction; in cortical-dominated sites, osseodensification burs may behave more as cutting instruments, with a limited densification effect and a potential increase in frictional stress or microdamage if compression is not adequately controlled. Therefore, in a low-density model with a thin cortical layer (type IV substrate), a conservative strategy consisting of a 0.3mm overpreparation was applied to reduce excessive insertion torque and peak compressive strain while preserving trabecular compaction at the osteotomy margins, which contributes to increased stiffness as measured by RFA. This balanced approach aims to optimize mechanical stability without unduly increasing the risk of compressive injury, a trade-off that is particularly relevant in clinical scenarios involving soft bone. Further research should evaluate how variations in cortical thickness modulate the efficacy and safety of osseodensification protocols and help define optimal osteotomy sizing across different bone types [9,11,12].  

This controlled overpreparation may partially explain the lower insertion torque in Group OD while still allowing a favorable stiffness response on RFA [9-12].

The opposite trends observed between torque and ISQ reinforce that these outcomes capture different biomechanical constructs. To further explore the relationship between the two stability metrics, within-group Pearson correlation analyses were performed. No relevant linear association was observed between insertion torque and ISQ in either experimental group. This finding supports the concept that insertion torque and ISQ reflect different biomechanical aspects of primary stability and should not be considered interchangeable parameters. Insertion torque reflects resistance during rotational advancement, while ISQ is more closely related to lateral stiffness and the boundary conditions at the implant neck. Therefore, clinical decisions should consider both metrics rather than relying on one measurement alone, particularly in low-density bone scenarios where stability may be marginal [13,14].

From a clinical standpoint, undersized osteotomy could be preferred when higher insertion torque is required, for example when aiming for immediate provisionalization under controlled conditions. Conversely, osseodensification may be beneficial when the objective is to improve stability as assessed by RFA and potentially favor bone compaction. The practical selection of a protocol may depend on the clinician’s strategy, the implant design, and the anatomical site [3,17-19].

The study has limitations inherent to an *in vitro* design, as it was performed using type IV polyurethane blocks with a dense cortical shell, a model widely employed in implant research because it provides high standardization and allows controlled comparisons between surgical protocols. The use of synthetic substrates reduces inter-sample variability that is common in cadaveric or animal bone (e.g., heterogeneity in trabecular architecture, cortical thickness, hydration, and storage conditions), thereby improving internal validity and the reproducibility of mechanical measurements such as insertion torque and resonance frequency analysis [15,16].

In this context, the present results should be interpreted as a mechanistic comparison between undersized osteotomy and osseodensification under stable low-density conditions, with minimized confounding factors.

Nevertheless, polyurethane models do not reproduce fundamental biological properties of living bone that are relevant to implant stability and clinical performance. Synthetic blocks cannot mimic bone remodeling dynamics, angiogenesis and vascular supply, cellular activity, or the inflammatory and reparative responses that occur after osteotomy and implant placement. In addition, the viscoelastic and anisotropic behavior of bone tissue, as well as the time-dependent changes in mechanical properties during healing, are absent in polyurethane [13,16].

Consequently, although polyurethane may reasonably simulate certain aspects of density and compressive behavior, it cannot replicate the biological environment that influences the transition from primary stability (mechanical) to secondary stability (biological). For this reason, the present findings cannot be directly extrapolated to predict osseointegration rates, marginal bone changes, or long-term clinical outcomes.

Beyond the substrate limitations, the generalizability of these findings is restricted by the experimental scope. Only one implant system (single macrogeometry and dimensions) was evaluated; therefore, the observed torque-ISQ profile may differ with other implant designs (e.g., conical vs cylindrical bodies, different thread pitch/depth, apex geometry, and surface characteristics) known to influence insertion torque and the stiffness of the implant-bone complex. Likewise, a single simulated bone density (type IV polyurethane with a cortical shell) was used, and variations in cancellous density and cortical thickness commonly found clinically may alter the mechanical response to undersizing or densification [8].

Finally, each technique was tested with one specific drilling sequence; different degrees of undersizing and different osseodensification parameters (bur sequence and rotational mode) could yield different stability patterns. Therefore, these results should be interpreted as applicable to the tested implant design and the specific preparation protocols under the present low-density model, and further studies are needed across different implant systems and substrate densities.

From a clinical standpoint, the observed differences between insertion torque and ISQ in the two protocols should be understood as reflecting different aspects of the implant-substrate interaction at time zero. *In vivo*, the net effect of these protocols may be modified by patient-related factors (bone quality and anatomy, local vascularity, systemic conditions), surgical variables (heat generation, irrigation efficacy, drilling pressure), and implant-related factors (macrogeometry and surface characteristics). Therefore, protocol selection in clinical settings should not rely solely on the mechanical outcomes reported here.

Standardized drilling sequences under controlled conditions can improve the reproducibility and accuracy of implant site preparation in experimental settings. [20] Despite these limitations, the present study provides a robust and reproducible framework to compare two commonly used preparation strategies and their effects on primary stability in low-density conditions. Future studies should validate these results in biological models and clinical trials, ideally including longitudinal measurements of stability (RFA over time), radiographic outcomes, and clinical endpoints to determine whether the mechanical patterns observed in polyurethane translate into clinically meaningful differences.

## Conclusions

Within the limitations of this *in vitro* study, undersized osteotomy produced higher insertion torque, whereas osseodensification produced higher ISQ values in a low-density polyurethane model. These findings suggest that the two protocols may be selected according to the clinical objective and the preferred stability metric.

## Figures and Tables

**Table 1:** Table 1: Primary stability outcomes by group (mean±SD).

**Outcome**	**Group OS (n=38)**	**Group OD (n=38)**	**p-value**
Insertion torque (Ncm)	30.66±1.71	26.58±2.63	<0.0001
ISQ	52.92±3.57	56.80±2.90	0.00000227

**Figure 1:**
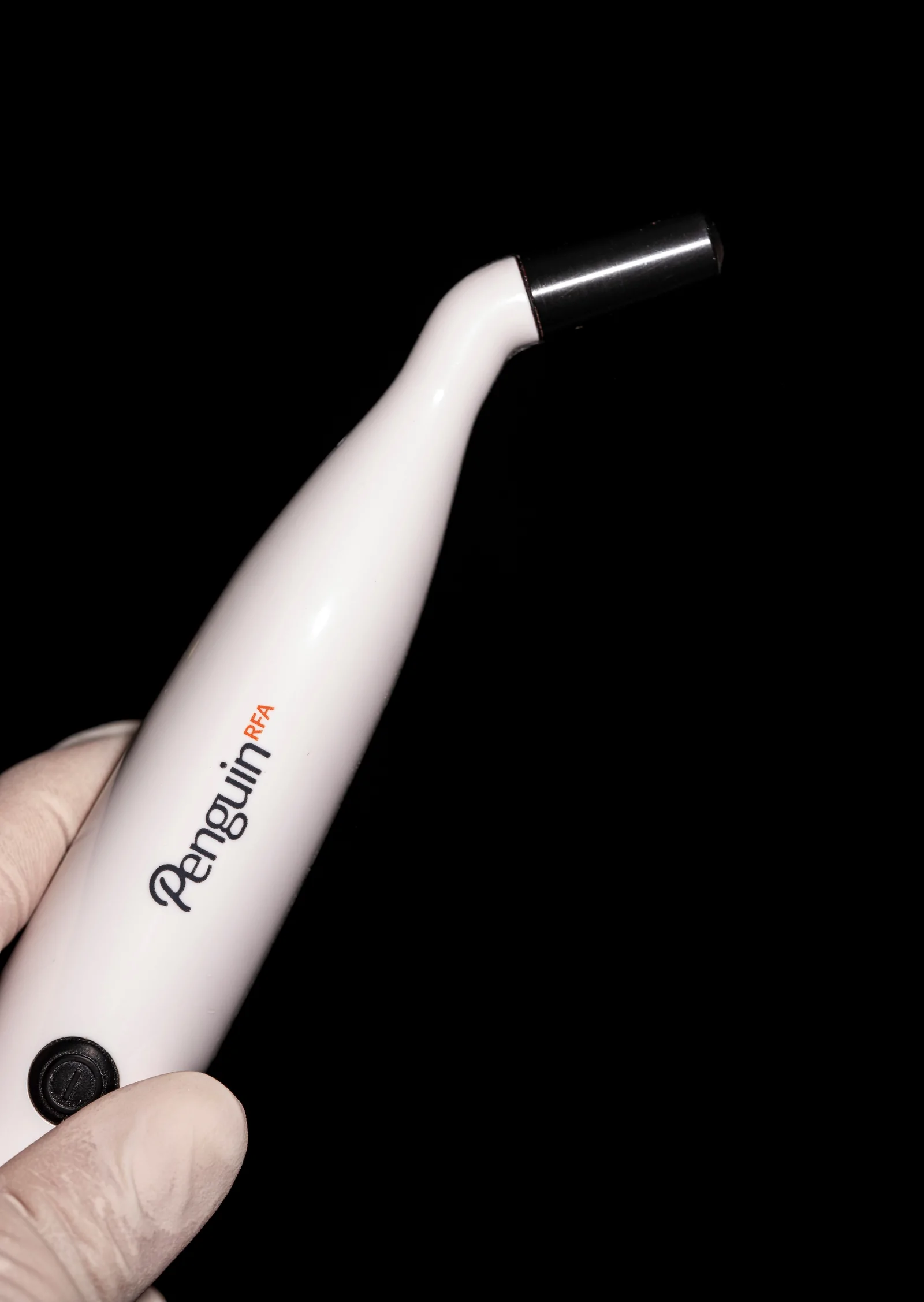
Fig. 1: Penguin® RFA device used to measure implant stability quotient (ISQ) in the present in vitro study.

**Figure 2:**
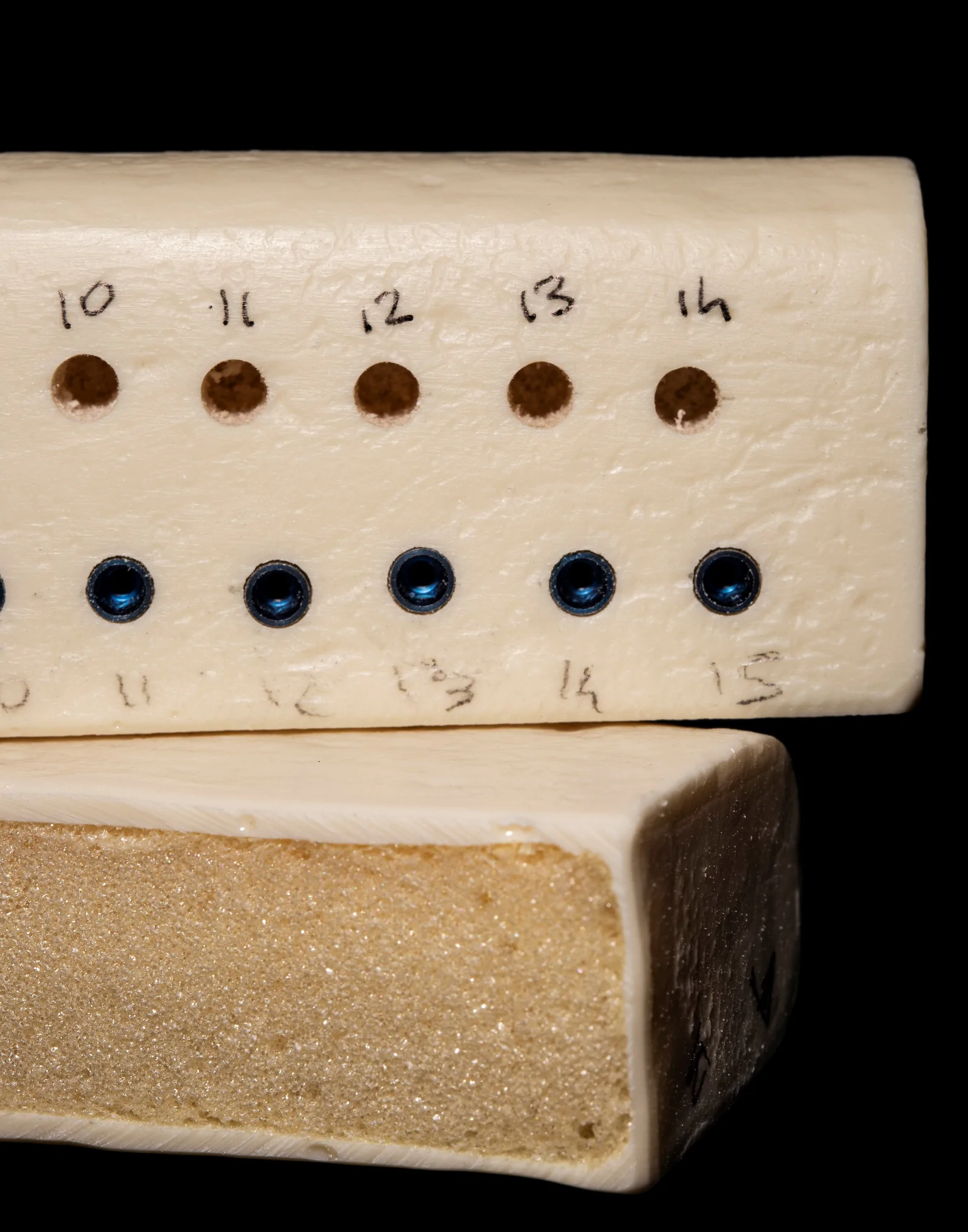
Fig. 2: Polyurethane block (simulated low-density bone) showing the standardized osteotomy sites and representative implants inserted during the experimental procedure.

**Figure 3:**
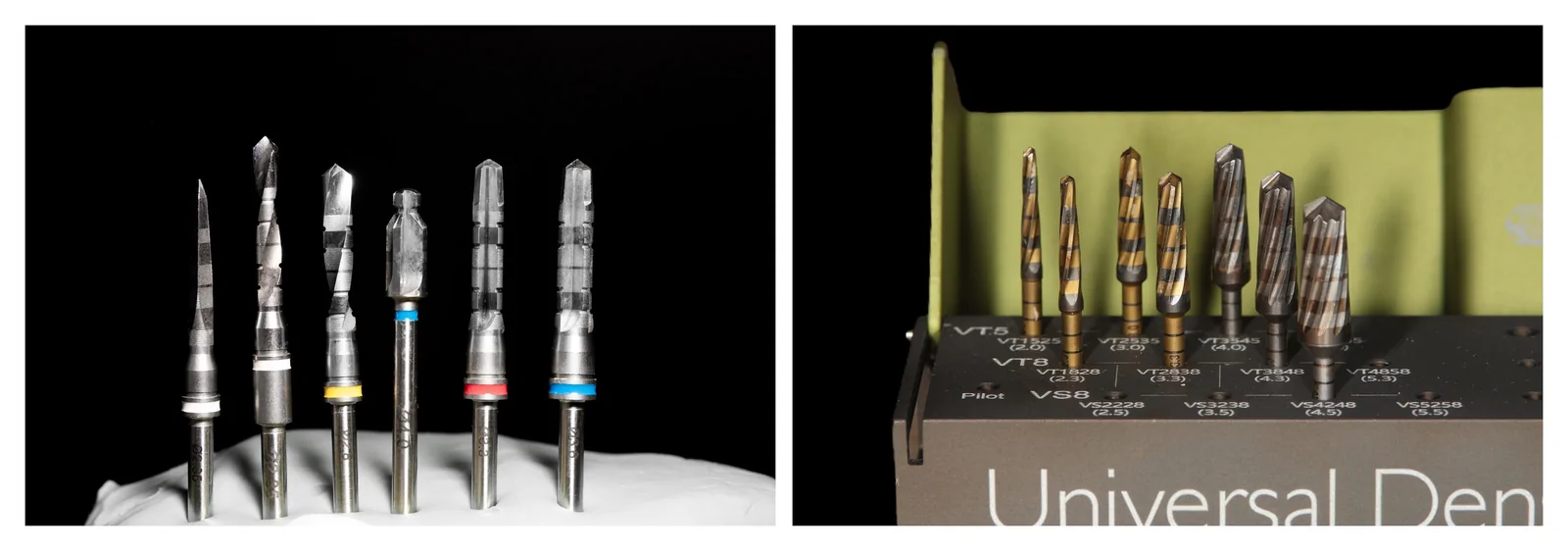
Fig. 3: Drilling instruments used for implant site preparation: (A) Conventional/undersized osteotomy kit (Group OS) and (B) Densah® burs for osseodensification (Group OD).

**Figure 4:**
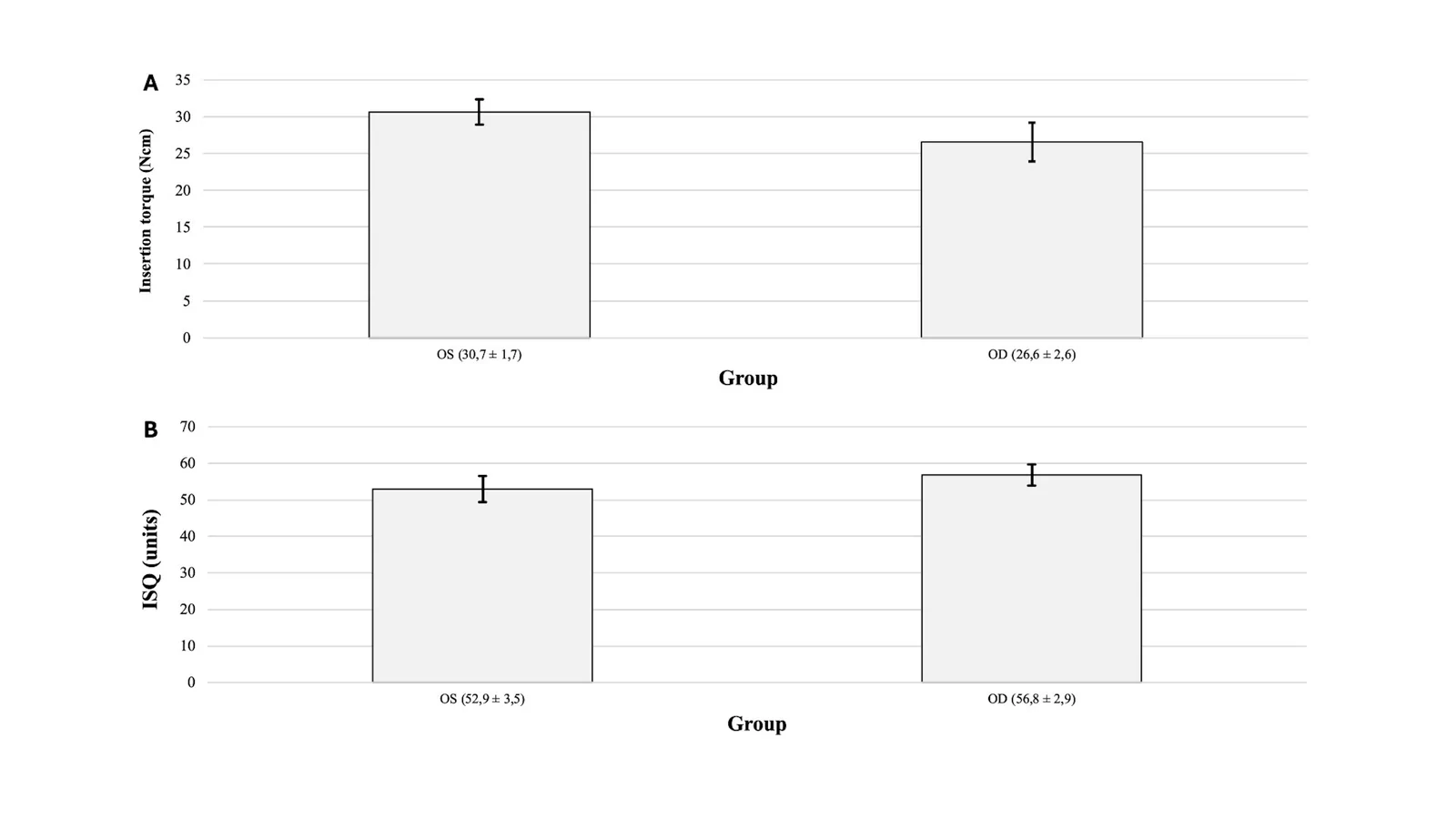
Fig. 4: Graphical representation of primary stability outcomes. (A) Mean insertion torque (Ncm±SD) for the undersized osteotomy (OS) and osseodensification (OD) groups. (B) Mean implant stability quotient (ISQ±SD) for both groups. Error bars represent standard deviation.

## Data Availability

None.
